# Immunity Profiling of COVID-19 Infection, Dynamic Variations of Lymphocyte Subsets, a Comparative Analysis on Four Different Groups

**DOI:** 10.3390/microorganisms9102036

**Published:** 2021-09-26

**Authors:** Mario Giosuè Balzanelli, Pietro Distratis, Gianna Dipalma, Luigi Vimercati, Orazio Catucci, Felice Amatulli, Angelo Cefalo, Rita Lazzaro, Davide Palazzo, Sergey Khachatur Aityan, Giancarla Pricolo, Antonella Prudenzano, Patrizia D’Errico, Rita Laforgia, Angela Pezzolla, Diego Tomassone, Alessio Danilo Inchingolo, Van Hung Pham, Donatello Iacobone, Giuseppe Mancusi Materi, Antonio Scarano, Felice Lorusso, Francesco Inchingolo, Kieu Cao Diem Nguyen, Ciro Gargiulo Isacco

**Affiliations:** 1 SET-118, Department of Pre-Hospital and Emergency, SG Giuseppe Moscati Hospital, 74100 Taranto, Italy; mario.balzanelli@gmail.com (M.G.B.); distratispietro@gmail.com (P.D.); oraziocatucci@live.it (O.C.); amatullidrfelice@libero.it (F.A.); ugemel@yahoo.it (A.C.); rita-lazzaro@libero.it (R.L.); davide.palazzo@gmail.com (D.P.); 2 Department of Interdisciplinary Medicine, University of Bari “Aldo Moro”, 70124 Bari, Italy; giannadipalma@tiscali.it (G.D.); luigi.vimercati@uniba.it (L.V.); francesco.inchingolo@uniba.it (F.I.); drkieukaren@gmail.com (K.C.D.N.); 3 Director Multidisciplinary Research Center, Lincoln University, Oakland, CA 94612, USA; aityan@lincolnuca.edu; 4 Department of Hematology, SS. Annunziata, 74100 Taranto, Italy; giancarla.pricolo@asl.taranto.it (G.P.); antonella.prudenzano@asl.taranto.it (A.P.); patrizia.derrico@asl.taranto.it (P.D.); 5 Department of Emergency and Organ Transplantation, University of Bari “Aldo Moro”, 70124 Bari, Italy; ritalaforgia@hotmail.it (R.L.); angela.pezzolla@uniba.it (A.P.); 6 Foundation of Physics Research Center, 87053 Celico, Italy; dietomoh@gmail.com; 7 Department of Microbiology, “Phan Chau Trinh” University of Medicine and Nam-Khoa Biotek, Ho Chi Minh 50000, Vietnam; van.pham@pctu.edu.vn; 8 SET-118, Department of Pre-Hospital and Emergency, BAT, 76121 Barletta, Italy; donato.iacobone@aslbat.it; 9 Anesthesia and Intensive Care Unit, Department of Emergency, University of Bari “Aldo Moro”, 70124 Bari, Italy; giuseppemancusi@live.it; 10 Department of Innovative Technologies in Medicine and Dentistry, University of Chieti-Pescara, 66100 Chieti, Italy; ascarano@unich.it; 11 American Stem Cells Hospital, Ho Chi Minh 70000, Vietnam

**Keywords:** SARS-CoV-2, cellular immunity, coronavirus, humoral response, broncho-alveolar lavage fluid (BALF)

## Abstract

Background: A novel coronavirus (SARS-CoV-2)-induced pneumonia (COVID-19) emerged in December 2019 in China, spreading worldwide. The aim of the present investigation was to evaluate the immunological response and the clinical subset of peripheral lymphocyte subset alteration in COVID-19 infection. Methods: the study was conducted on four different clinical groups (*n* = 4; total *n* = 138). Each individual was assigned to different groups based on specific criteria evaluated at the admission such as fever, dyspnea, arterial blood gas analysis (ABG), oral-nasopharyngeal swab/RT-PCR, and thoracic CT-scan. Treatment was performed only after blood samples were collected from each patient (PP and PP) at day 1. The blood samples were analyzed and tested the same day (CBC and Flowcytometry). The positive–positive group (PP *n* = 45; F = 18/ M = 27; median age = 62.33), comprised individuals affected by COVID-19 who showed fever, dyspnea (ABG = pO2 < 60), confirmed positive by oral-nasopharyngeal swab/RT-PCR and with CT-scan showing ground-glass opacities. The negative–positive (NP; *n* = 37; F = 11/M = 26; median age = 75.94) or “COVID-like” group comprised individuals with fever and dyspnea (ABG = pO2 < 60), who tested negative to nasopharyngeal swab/RT-PCR, with CT-scans showing ground-glass opacities in the lungs. The negative–affected group (NA; *n* = 40; F = 14/M = 26; median age = 58.5) included individuals negative to COVID-19 (RT-PCR) but affected by different chronic respiratory diseases (the CT-scans didn’t show ground-glass opacities). Finally, the negative–negative group (NN; *n* = 16; F = 14/M = 2) included healthy patients (NN; *n* = 16; median age = 42.62). Data and findings were collected and compared. Results: Lymphocytes (%) cells showed a decline in COVID-19 patients. The subsets showed a significant association with the inflammatory status in COVID-19, especially with regard to increased neutrophils, T-killer, T-active, T-suppressor, and T-CD8+CD38+ in individuals belong to the either COVID-19 and Covid-like NP group. Conclusions: Peripheral lymphocyte subset alteration was associated with the clinical characteristics and progression of COVID-19. The level of sub-set cells T-lymphocytes (either high or low) and B-lymphocytes could be used as an independent predictor for COVID-19 severity and treatment efficacy.

## 1. Introduction

The novel Chinese COVID-19 disease rapidly spread throughout the world, the genome-wide sequencing obtained from samples of broncho-alveolar lavage fluid (BALF) confirmed it to be a distinct type of the β-coronavirus associated with human severe acute respiratory syndrome (SARS) and Middle East respiratory syndrome (MERS) [1]. During COVID-19 infection, alterations of T and B-lymphocytes were seen, and correspondingly, subsets suggested a potential association with the Sars-CoV-2 pathogenic mechanism [1]. Results indicated general disturbances within peripheral lymphocytes in COVID-19 patients; however, the abnormal status of their subsets remains to be clarified [2,3,4,5,6,7]. The human immune system has evolved to allow the host to co-exist with an enormous quantity of pathogens, microorganisms and microbes that are themselves constantly evolving. Both innate and adaptive arms of immune system are able to detect, distinguish, and eliminate pathogens while recognizing self-agents that are potentially dangerous for the host [8]. It is well known that the SARS-CoV-2 triggers multiple immune reactions simultaneously and often conflictual. It has been observed that both T and B lymphocyte exhaustion followed prolonged increased inhibitory signals on specific receptors secondary to an augmented apoptosis process involving modulating sub-group lymphocytes such as CD45RA+ (naïve) either CD4 or CD8 within the memory compartments [8,9,10,11,12,13,14]. However, data showed that convalesced individuals revealed more virus-specific CD4 T cell responses than virus-specific CD8 T cell responses, though pre-existing CD4 T cell responses to other coronaviruses also are found in a subset of subjects in the absence of SARS-CoV2 exposure [9]. Though it remains unclear how these phenomena should be interpreted in the context of COVID-19 lymphocytopenia, it can be assumed that cytotoxins, suppressor T cells, and macrophages (M1) GM-CSF (CD80–86) may all be factors in secondary uncontrolled IL-6 responses while decreasing T cells (Treg), gamma/delta T cells (T-ɣδ), and CD4 immune modulating responses [9,15,16,17]. In addition, more attention should be placed on B cells and B sub-groups pleiotropism in COVID-19 infection, as both virus and virus-infected cells may also strike the B lymphocyte production centers, which explains the reduced presence of specific antibodies against the virus in heavily affected COVID-19 patients. The neutralizing antibodies are highly effective when viruses are present in serum or on moist surfaces such as the respiratory and gastro-intestinal tracts [18,19,20,21,22,23,24]. The immune-globulins like the IgG, IgM, and IgA are all able to exert antiviral activity, deactivating viruses through methods such as blocking virus-host cell interactions, recognizing viral antigens on virus-infected cells, or by complement-mediated lysis [10,11,24,25,26,27,28]. However, regardless the quantity of circulating antibodies, the occurrence of memory T cells and B-lymphocytes due to SARS-CoV-2 stimulation is crucial for long-term immunity. In this scenario, the attendance of active T helper cells is suggestive of normal configurations of humoral immune response and the formation of specific memory B cell clusters capable of initiating responses against further reinfection [16,23,24,29,30,31,32,33,34,35,36,37,38,39]. Indeed, the presence of neutralizing antibodies has been revealed to be a good resource to control SARS-CoV2 infection either in vitro or in vivo, though not all patients who recover from COVID-19 show enough neutralizing antibodies to be used as anti-COVID-19 therapy, suggesting a complex relationship between humoral and cellular response in COVID-19 pathogenesis [30,31,32,40,41,42]. This was a retrospective observational study including hospitalized adult patients with SARS-CoV-2 infection. The study was approved by the local Ethical Committee of SG Moscati Hospital of Taranto-Italy (prot. N. 36805, approval date: 16 June 2020) and was conducted by the 118 Pre-Hospital and Emergency Unit. The main aim was to describe the clinical implication of peripheral lymphocyte subset alteration in COVID-19 performed via a comparative analysis on the different groups. The analyzed immune profile on hospital admission consisted of differential blood cell counts (total white cell count, neutrophils, lymphocytes), subtyping of lymphocytes by flow cytometry (on the day of admission) that included the basic panel (CD3+, CD19+, CD4+, CD8+, CD4 Naïve CD45RA, CD8 Naïve CD45RA, CD16+56+, T killer, T-active CD3+DR, CD8+CD38+DR+, CD8−cytotoxic CD57−, CD8-suppressor CD57+, CD8+CD38+DR+, T-reg CD4+CD25+high, monocytes, monocytes CD14+/CD16+), and phenotyping of B-cell.

Here, each data showed positive and negative correlations. The results revealed a distinctive pattern of cytokines showing a positive correlation with age and sex (Figures 2–7). The considered set of cytokines also revealed an association with hospitalization time, age and sex (data not included). As expected, the cytokine signatures associated with PP and NP groups were partially overlapping. These shared cytokines logically included molecules that have been implicated in COVID-19 pathogenesis such as IL-6, eGFR, vitamin D3, and fibrinogen, as well as molecules that are more generally associated with inflammation/infection, such as VES, D-dimer, PCR, and iron [39]. Interestingly, white cells, neutrophils, lymphocytes, monocytes, NK and T/B lymphocytes subsets were predominately increased or decreased in older patients (Figures 2–7). To further associate the identified profiles with the clinical evaluation, the percentage plasma concentrations of the aforementioned age-dependencies were compared in patients with different lung pathologies and with healthy ones, confirming that the cell-mediated immune response was significantly up-regulated in COVID-19 cases, as compared to other patients.

## 2. Materials and Methods

### 2.1. Study Population

The laboratory panel and clinical/radiological data were obtained after signing of the informed consent by all patients recruited in the present investigation, in accordance to the Declaration of Helsinki. The study was approved by the Internal Committee of SG Moscati Hospital of Taranto. The subjects were male and female with an ages ranging from 21 to 95 years old (*n* = 138). All patients underwent a screening process for COVID-19 and were admitted into the 118 Emergency and Pre-Hospital Department of SG Moscati Hospital of Taranto City during the period between 14 April to 25 September 2020. The study data on lymphocyte subsets was obtained from four different groups. First group, the study group (PP, *n* = 45) were positive for COVID-19 (as confirmed by RT-PCR, oral-nasopharyngeal swab), had thoracic CT-scans showing ground-glass opacity, fever, dyspnea, and pO2 < 60% by ABG analysis. The second group, a control group (NP, *n* = 37), tested negative for the Sars-CoV-2 virus (RT-PCR) and had ground-glass CT-scan, fever, and pO2 < 60% by ABG. The third control group (NA, *n* = 40) tested negative by both RT-PCR and CT-Scan but were affected by different respiratory diseases. The fourth control group (*n* = 16) comprised healthy individuals (NN). The basic patient information is shown in Table 1.

Samples were collected by withdrawing peripheral blood using specific vacutainers with EDTA (BD Becton, Dickinson and Company, Franklin Lakes, NJ, USA). The blood samples from PP, NP, NN, and NA were obtained at the time of hospital admission and/or visit (T0). Different hematology, lab parameters, and flow-cytometry were processed the same day.

The COVID-19 nucleic acid detected by naso-oralpharyingeal swabs was collected at the time of patient admission. The RT-PCR confirmed the presence of Sars-CoV-2 in the PP group while being negative in all the other groups NP, NA, and NN. The CT-scan was performed on PP, NP, and NA patients only.

### 2.2. RT-PCR and Nucleic Acid Detection

The COVID-19 kit (Real-Amp kit) was provided by OSANG-Healthcare of Korea Co. Ltd. (Seoul, Korea). The COVID-19 nucleic acid test detects the N and 1ab genes, and the quality of nucleic acid extraction was monitored by using an internal standard. A reaction-mixture, a probe mixture, a positive control, and one negative quality control were used for each set. When two genes were amplified and the cycle threshold value was detected under the detection limit, the result was considered to be positive. To reduce variabilities and bias, the whole samples preparation, nucleic acid extraction, and dilutions were carried out in a different laboratory located in the same city at the SS Maria Annunziata Hospital of Taranto City (Apulia, Italy). The viral RNA was extracted using the GeneFinder™ COVID-19 PLUS RealAmp Viral RNA kit (OSANG Healthcare Co., Ltd., Anyang, Korea), as per manufacturer’s instructions from naso-pharyngeal swab and sputum samples. COVID-19 PLUS Reaction Mixture and 5 μL of COVID-19 PLUS Probe Mixture, was used to prepare the RT-PCR master mixture, following manufacturer’s instructions for each single set. Enough master mixture was prepared for all the reactions plus extra amounts to prevent possible pipetting error (total master mixture number = *n* sample + 1 positive control + 1 negative control + 1 extra). Successively, 15 μL of RT-PCR Master mixture was poured into each PCR tube or optical 96 well plate, 5 μL of sample RNA was added into the corresponding PCR tube/well-plate for amplification and each well was mixed by pipetting. 5 μL of positive control and negative control was placed into single PCR tube and placed for testing into the real-time thermal cycler for amplification.

### 2.3. Flow Cytometry Hematology Parameters

Blood samples were collected and processed the same day. The blood routine indicators included different subgroups: WBC, lymphocytes, neutrophils, T cells (different phenotypes), and B cells. The antibodies used were as follow: mature CD3 (FITC BD Bioscience, Franklin Lakes, NJ, USA), CD19 (APC, BD Bioscience), CD16–56 (PE, BD Bioscience), CD4 (Pe-Cy7, BD Bioscience), CD8 (APC-H7, BD Bioscience), HLA-DR+ (APC-H7, BD Bioscience), CD57 (FITC BD Bioscience), CD38 (PE-Cy7, BD Bioscience), CD25 (PE, BD Bioscience), and monocytes CD14 (FITC, BD Bioscience) (BD Bioscience, NJ, USA). All analyses were conducted using FSC/SSC (Becton Dickinson immunocytometry system) calibrated with CaliBRITE brand beads and AutoCOMP software (Caron Eng., Wells, ME, USA)

USA. For the standard technique a minimum of 50,000 to 100,000 total events were acquired, with LYSYS II software (BMSR Biomedical Simulations Resource, Los Angeles, CA, USA) and CD45/Side SCATTER (SSC) used to establish an analysis gate that included at least 95% of lymphocytes and no more than 5% of monocytes in the sample. Markers for determining positive and negative cell results were set by LYSYS II software with conjugated antibodies of irrelevant specificity as negative controls. The gate sequence strategy was as follows: (i) flow stability gating (time); (ii) cell selection with; (iii) doublet exclusion via plot FSC-A/FSC-H (Area vs Height gating strategy). Data were displayed as two-color dot plots (FL1 versus FL2). All samples were processed within 24 h after staining. The absolute numbers of cells (10^6^/liter) for each lymphocyte subset were calculated by multiplying the corrected relative value by the total lymphocyte count. The analysis procedure followed standard isolation protocols which consisted of direct labelling followed by erythrocyte lysis, washing, and reading.

### 2.4. Statistical Analysis

Statistical significance was determined using Student’s *t*-test with 95% confidence comparisons between the four groups. Where indicated, the *z* score of percentage of marker expression was calculated as follows: *z* = (*x* − μ)σ, where *x* is the raw score, μ is the mean of sample distribution, and σ is the SD (95%). For categorical comparisons, Fisher’s exact test was used. Significant PhenoGraph clusters (*p* ≤ 0.05) were determined by chi-squared goodness-of-fit tests comparing the relative abundance of each categorical group in each individual PhenoGraph cluster relative to input.

A standard model for describing the evolution of the infected cases by viruses can be constructed as follows:

(1) dxdt=E(x)∗I(x), with E(x)=λ∗x, I(x)=1−x^b

where *x*(*t*)≡*N*(*t*)/*N*_max_, *x*_0_≡*N*_0_/*N*_max_; *N*(*t*) is the number of total infected cases evolved from the initial *N*_0_≡*N*(0) cases, *N*_max_ is the maximum possible number of infected cases; λ is the exponential growth rate, and becomes clear for *x*(*t*) << 1 where *I* is negligible, leading to:

(2) x=x (0)∗exp(λt)or N(t)=N (0)∗exp(λt), for x≪1 where I (x)~1

The function of negative feedback, *I,* models the factors that flattens the curve, such as, the measures taken against spreading. While these factors are not affecting the exponential growth rate λ, they become more effective as the number of cases increases, getting closer to *N*_max_; exponent *b* controls the effectiveness of these factors; strict {loose} measures correspond to smaller {larger} values of *b*.

## 3. Results

**Figure 1:**
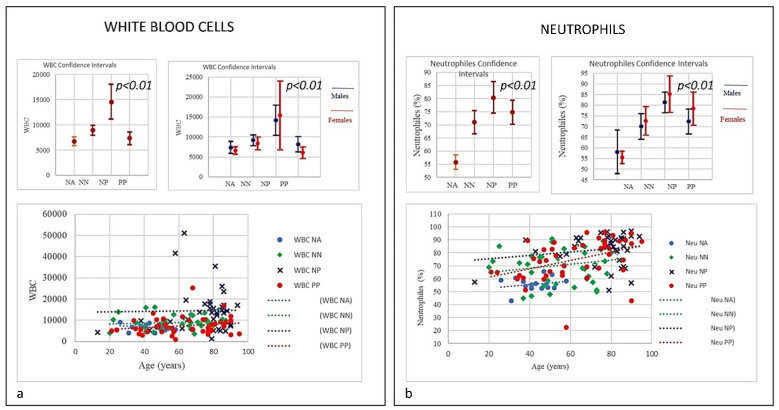
(**a**) Frequencies of white blood cells (WBC). The differences in WBC count and neutrophils between PP patients and controls NP, NN, and NA (age and sex) were statistically significant (*p* = 0.01), the trend indicated that PP patients showed the lowest level of WBC, very close to NN group compared to NP group. (**b**) Neutrophils in COVID-19 patients and controls (NP, NN and NA). The neutrophils trend in PP, NP, and NN groups was increasing with age. Both PP and NA started from a lower range compared to NP. PP and NP shared the highest level with age.

**Figure 2:**
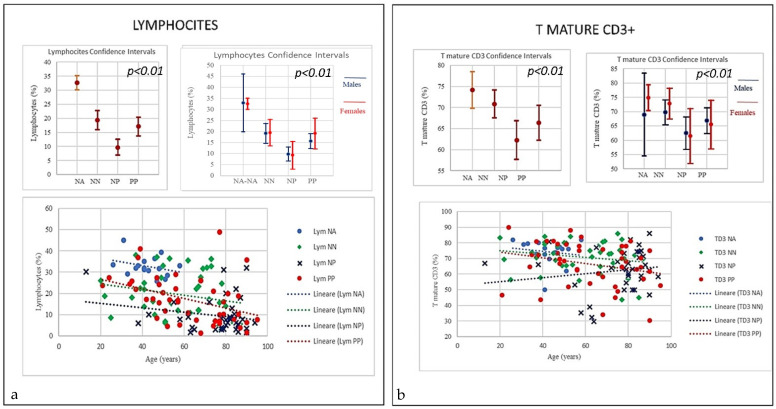
(**a**) Frequencies of Lymphocytes and T mature CD3 in COVID-19 patients and controls (NP, NN and NA). There was a decrease in lymphocytes in all PP, NP and NN, however both PP and PP the trend started from higher range whilst the NP the range started lower. Nevertheless, at certain age the, around 80, we observed the worst decay. (**b**) Frequencies of T-mature CD3 in COVID-19 patients and controls (NP, NN and NA). A negative correlation was observed between the age and the percentage of T mature CD3 between PP and NP (*p* = 0.01); the PP patients T CD3 decline with age starting from higher range while in NP that line increase with age but starting from lower range (*p* < 0.001).

**Figure 3:**
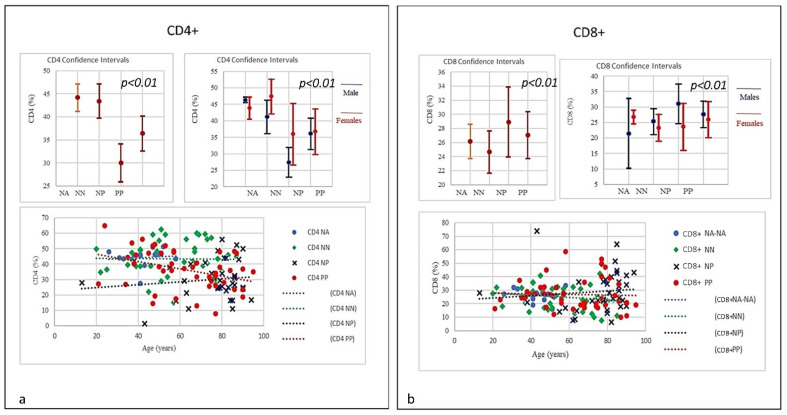
(**a**) Frequencies of CD4+ lineage in COVID-19 patients and controls (NP, NN and NA). A negative correlation was observed between the age and the percentage of T CD4 between PP and NP. PP patients T CD4 levels from a normal range tend to decline with age, conversely NP showed an increasing trend starting from lower range level and increasing with age (*p* < 0.001). (**b**) Frequencies of CD8+ lineage in COVID-19 patients and controls (NP, NN and NA). The CD8 did show a quantitative increase correlated with age in NP group (the older the higher). Conversely a slightly quantitative decrease in PP and NN groups was seen correlated with age (the older the lesser).

**Figure 4:**
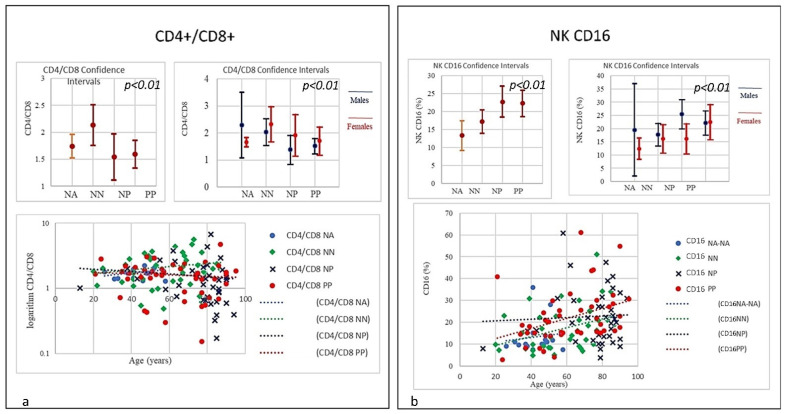
(**a**) Frequencies of CD4/CD8 ratio in COVID-19 patients and controls (NP, NN, and NA). The PP and NP groups showed a similar low trend (<1) compared to the control groups NN and NA (*p* < 0.001). A low or inverted CD4/CD8 ratio is associated with abnormal immune activity, immune senescence, and chronic inflammation, conditions typical of HIV populations. The prevalence of an inverted CD4/CD8 ratio increased with age. (**b**) Frequencies of NK CD16 lineage in COVID-19 patients and controls (NP, NN, and NA). A strong correlation was observed between age and the NK increase in the PP group: the older the higher; the NP group showed a lesser increase, though the starting NK levels were significantly higher than PP individuals (*p* < 0.01).

**Figure 5:**
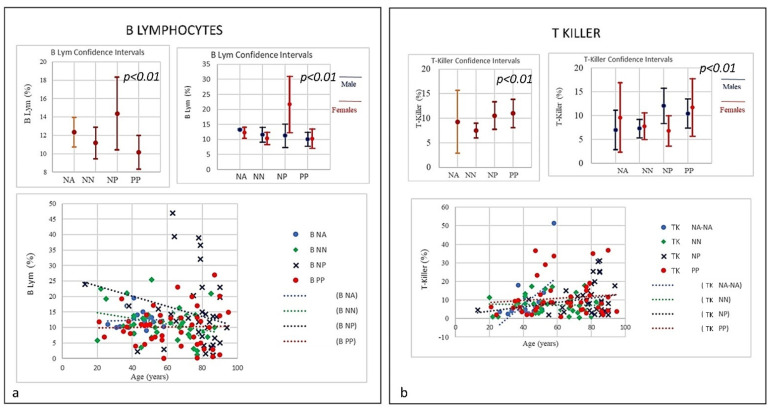
(**a**) Frequencies of B Lymphocytes in COVID-19 patients and controls (NP, NN, and NA). Data revealed a linear decrease in levels of B-lymphocytes. PP individuals at time of the admission showed a low level of B lymphocytes, though both NP and NN individuals started with a higher level of B cells showing a decrease correlated with the age. (**b**) Frequencies of T- and N killer Lymphocytes in COVID-19 patients and controls (NP, NN and NA) there was an increase in the three groups PP and NP (high) and NN (low) suggestive of active capability of immunity to respond as killer T cell recognizes and kills virus-infected cells because of the viral antigen on its surface.

**Figure 6:**
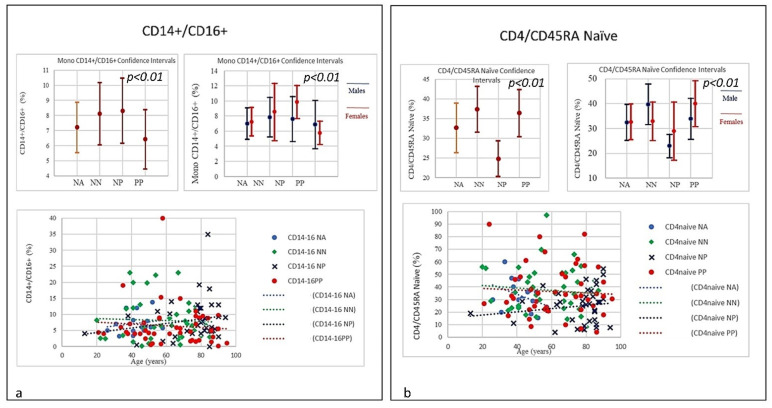
(**a**) Frequencies of CD14+CD16+ Monocyte lineages ratio in COVID-19 patients and controls (NP, NN, and NA). A significant increase in quantitative level of CD14+CD16+ monocytes correlated with age was observed. The increased level in the elderly may indicate they have undergone a senescence process and therefore they also secrete proinflammatory factors in response to low-intensity antigenic stimuli. A slight quantitative decrease in the PP group, which was not correlated with age, was also observed. However, the levels of both intermediate CD14+ and CD16+ as well as non-classical monocyte Neutrophils and NK cells were significantly elevated in NP patients. (**b**) Frequencies of CD+CD45RA Naïve level in COVID-19 patients and controls (NP, NN, and NA). The graphic shows a slight quantitative decrease in CD4 naïve in the PP group without correlation to age; however, there is a quantitative increase in CD4 naïve correlated to age in NP individuals. Notably, the starting level of CD4 naïve in the NP group was significantly lower than the one seen in PP group. Though the mechanism is yet to be clarified as, unlike age related changes in other T cell subsets, the stable level of T memory cells in peripheral blood may support CD4 naïve cells for long-term immunological memory, while their importance may increase together with ageing. However, higher morbidity and mortality following infections, particularly influenza, has been observed in elderly individuals.

**Figure 7:**
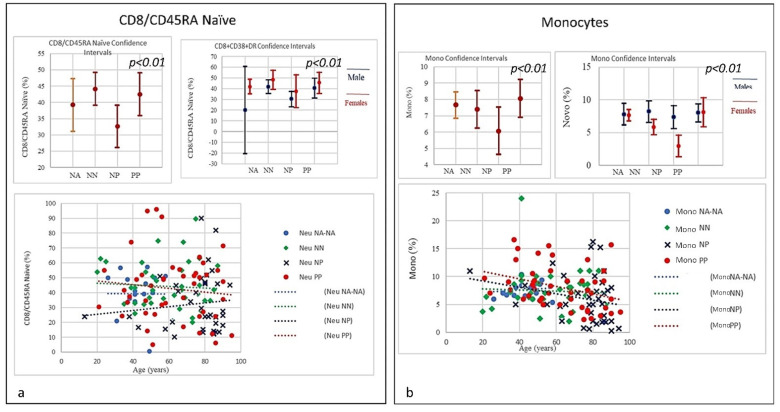
(**a**) Frequencies of CD8+/CD45RA Naïve level in COVID-19 patients and controls (NP, NN, and NA). The graphic shows a slight quantitative decrease in CD8 naïve for PP without correlation to age, while a quantitative increase in CD8 naïve in correlation to age in NP individuals was observed, CD8 naïve in the NP group started to increase from a lower level than PP. Though the mechanism is yet to be clarified as unlike age related changes in other T cell subsets, the stable level of T memory cells in peripheral blood may support CD8 naïve cells for long-term immunological memory, while their importance may increase together with ageing. (**b**) Frequencies of Monocytes percentage in COVID-19 Patients and Controls (NP, NN, and NA). The graph shows a decline in both PP and NP as age increases, mainly due to a general decline of naïve lymphocytes in the bone marrow and thymus as well as the expansion of incompetent memory lymphocytes.

**Figure 8:**
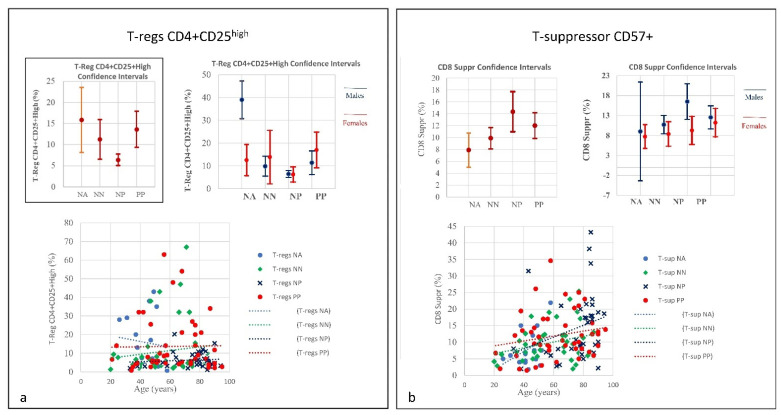
(**a**) Frequencies of T-regs CD4+CD25^high^ in COVID-19 patients and controls (NP, NN and NA) (CI 95%). Frequencies of T-regs in COVID-19 patients indicate a quantitative linearity without correlation to age. Similarly, the NP group showed the lowest quantitative level of T-regs with a correlation to age. The NN group was observed in between the PP and NP groups. The prominent point of severe COVID-19 is determined by an uncontrolled pro-inflammatory cytokine activity, the so-called “cytokine storm” that is in part due to deficiencies in immune regulatory mechanisms of T-regs key regulators of immune responses which are essential in the maintenance of immune homeostasis. The T-regs modulate the antiviral defense at the early stage of infection and reduce inflammation at the late stage of COVID-19. Right: Frequencies of T-suppressor CD57+ in COVID-19 Patients and Controls (NP, NN and NA) (CI 95%). (**b**) Frequencies include totality and divided by gender. T-suppressor quantitative increase in PP and NP correlating to age. In the NP group one can see that, quantitatively, the NP showed the highest rate of T-suppressors in elderly patients; the T-suppressors also increase in NN but in this case there is no correlation to age. The graphic indicates greater uniformity among the individuals. The original function of T-suppressor is blocking the activity of some other types of lymphocytes, to keep the immune system from becoming over-active. T-suppressor CD8+CD57+ also plays a significant role in various diseases or conditions, associated with chronic immune activation such as cancer, chronic intracellular infections, and some chronic pulmonary diseases.

We compared all laboratory sub-set lymphocyte indices for the 138 patients. To confirm the hypothesis that the recovery from COVID-19 may relate to well equilibrated lymphocyte immunity responses, we evaluated the signatures of the different subsets. To that end, we collected fresh samples from each individual in the four groups PP, NP, NA, and NN, and attempted to define the COVID-19 disease based on the different lymphocyte subsets parameters. Deteriorating rates in PP and NP patients occurred at similar frequencies, and very differently from the NA and NN groups (Figure 1, Figure 2, Figure 3, Figure 4, Figure 5, Figure 6 and Figure 7). To characterize the involvement of different lymphocyte subsets we developed a staining panel dedicated to identifying distinct T subsets and B cells. Percentage fits were obtained for all lymphocyte subsets listed in Table 1, we evaluated the distribution of each cluster across the four groups (PP, PN, NA, NN) and we compared the levels of each. The top 18 complementarities determining T, B-lymphocytes, and respective subtypes were different across four groups. Outcomes showed either reductions or over expressions in count and proportion of neutrophils, lymphocytes, NK cells, B lymphocytes (CD19), CD4, CD8, CD8 suppressor, T-reg CD4+CD25+^high^, CD4/CD45RA+ Naïve, CD8/CD45RA+ Naïve, T killer, and CD8+CD38+DR+ (Figure 1, Figure 2, Figure 3, Figure 4, Figure 5, Figure 6, Figure 7 and Figure 8).

**Figure 9:**
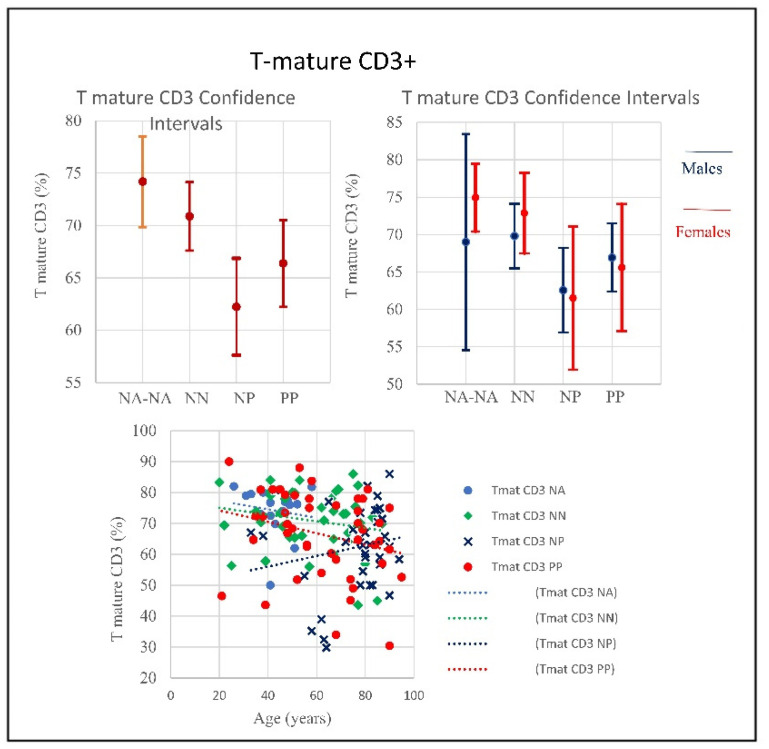
Frequencies of T-mature CD3+ in COVID-19 patients and controls (NP, NN, and NA) (CI 95%). Frequencies include totality and divided by gender. The T-mature quantitative trend decreased in PP and NA, showing no correlation with age, conversely the graphic shows that the NP trend tends to increase, correlated with age. Low T cell count usually indicates conditions affecting the immune system or lymph nodes due to viral infections such as influenza, and aging.

**Figure 10:**
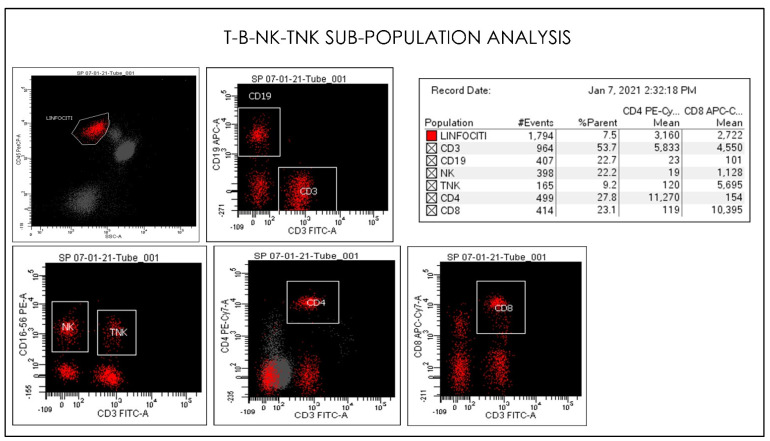
Gating Strategy for lymphocytes CD4, CD8, CD3, CD19, NK, and TNK. quantification. Comparative Dot plots of CD3 on X-axis vs. CD4, CD8, CD3, CD19, NK, and TNK. A doublet exclusion gate based upon (FSC-A vs. FSC-H) was utilized to gate on singlet cells, and within this singlet cell gate a lymphocyte gate was drawn in a dot plot of FSC-A vs. SSC-A. The instruments were set using one set of target values as described in the Method section of this paper. Percentages of positive cells were determined after gating on CD45 positive cells following the doublet exclusion.

**Figure 11:**
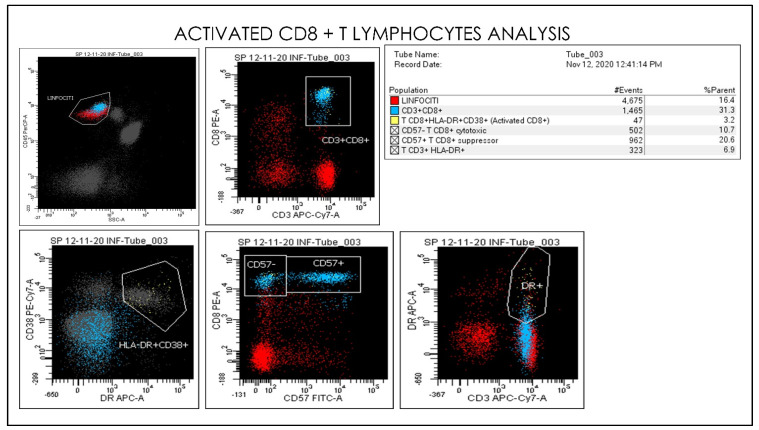
Gating Strategy for Monocytes CD33, CD45, CD16, CD56, and CD14 quantification. A doublet exclusion gate based upon (FSC-A vs. FSC-H) was utilized to gate on singlet cells, and within this singlet cell gate a lymphocyte gate was drawn in a dot plot of FSC-A vs. SSC-A. Comparative Dot plots of CD3 and CD57 on the X-axis vs. CD33, CD45, CD16, CD56, and CD14. The instruments were set using one set of target values as described in the Method section. Percentages of positive cells were determined after gating on CD45 positive cells following the doublet exclusion.

**Figure 12:**
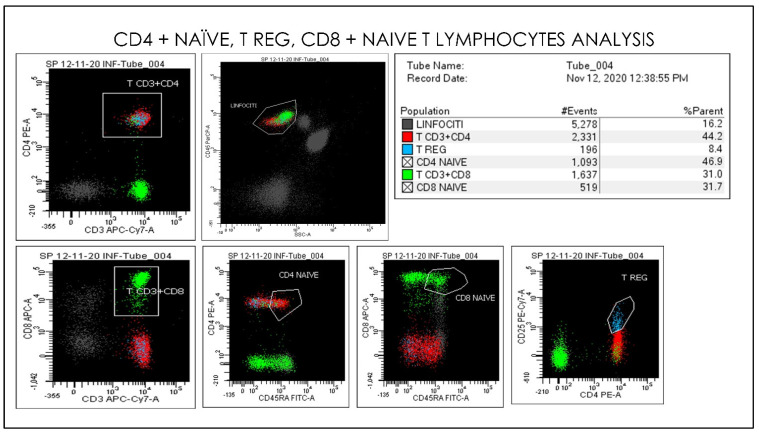
Gating Strategy for Lymphocytes, CD4 naïve, CD8 naïve, T reg, CD3+CD8+ quantification. A doublet exclusion gate based upon (FSC-A vs. FSC-H) was utilized to gate on singlet cells, and within this singlet cell gate a lymphocyte gate was drawn in a dot plot of FSC-A vs. SSC-A. Dot plots of CD3 and CD45 on the X-axis vs. CD33, CD45, CD16, CD56, and CD14. The instruments were set using one set of target values as described in “Method”. Percentages of positive cells were determined after gating on CD45 positive cells following the doublet exclusion.

**Figure 13:**
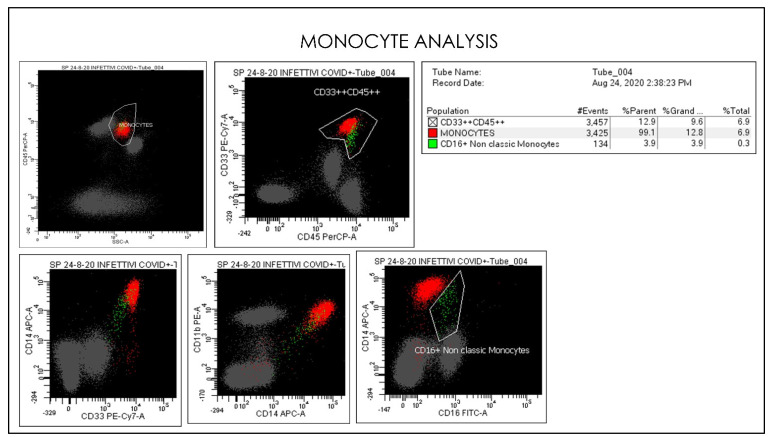
Flow cytometry gate-strategy show activated monocytes subsets and CD16 non-classic monocytes. In the conventional gating strategy is represented classical monocytes and non-classical monocyte populations based on CD14+CD16+ monocytes expression.

In the study, relevant traits as lymphopenia (PP = 64%), low level of B-lymphocytes (PP = 60%), low level of T-reg CD4+CD25+^high^ (PP = 37.8%) and high level of T killer cells (PP = 73.3%), high level of CD8+CD57+ suppressor (PP = 64.44%), high level of CD8+CD38+DR+ (80%), and monocytes (PP = 28.9%) were seen in COVID-19 patients. A picture suggestive of profound damage to the immune system during the course of SARS-CoV-2 infection (Table 1) [18,19]. Differences were detected using Student’s t-test between the four groups examining the cohort with low-expression and high-expression lymphocyte subsets including lymphocyte, neutrophils, and both T and B-cells and a selection are illustrated in Figure 1, Figure 2, Figure 3, Figure 4, Figure 5, Figure 6 and Figure 7 using the single exponential fit for purpose of comparison. Cell count (%) follows the lymphocyte subsets either increasing or simply sloped with age and sex. Best-fit, 95% confidence interval lines, were fitted for the PP cohort (dot red line), the NP (dot blue line), lung disease patients/NN (non-Covid-19, dot green line) and healthy participants/NA (dot sky blue line) for comparison. A trend toward higher counts (%) (St.d. 19.42; mean 62,33; CI 95%) of T-killer cells (St.d. 9.57; mean 10.98), CD8+CD38+DR+ (St.d. 11.97; mean 8.52), CD8+CD57+ suppressor (St.d. 7.21; mean 12.00), and monocytes, and absolute low counts of B cells and T-reg CD4+CD25+^high^ (St.d, 6.12, mean 10.16; St.d. 14.33; mean 13.44 respectively), were calculated in the PP group compared to NN and NA individuals (not NP). Significantly higher counts (%) (St.d. 16.67; mean 75,41; CI 95%) for neutrophils (St.d. 17.93; mean 80.41), NK-cells (St.d. 12.93; mean 22.68), monocytes CD14+CD16+ (St.d. 6.43; mean 8.30) and activated CD8 T-cells (St.d. 10.13; mean 17.89), were associated with decreasing of lymphocytes (St.d. 8.22; mean 9.68), CD4 (St.d. 12.45; mean 29.98), CD8 (St.d. 14.89; mean 28.90), CD4/CD45RA+ Naïve (St.d. 13.62; mean 24.84), CD8/CD45RA+ Naïve (St.d. 19.56; mean 32.60), CD4/CD8 ratio (St.d. 1.28; mean 1.54) in the NP cohort, once compared to NN and NA cohorts (Table 1). Based on this distribution, we observed marked differences in leukocyte count, neutrophils, monocytes, and different subsets between PP patients and controls NP, NN, and NA (age and sex) were statistically significant (*p* = 0.05). A significant lower quotient of CD4, T-mature CD3, CD4 naïve, CD8 naïve and monocytes CD14+CD16+ was observed in the PP group compared to the other groups (R = −0.34, *p* = 0.009) (Table 1, Figure 2 and Figure 3, Figure 6 and Figure 7). Interestingly, PP patients also showed the lowest decreased level of B-lymphocytes when compared to NP, NN, and NA (Table 1, Figure 2, Figure 3, Figure 4, Figure 5, Figure 6, Figure 7, Figure 8 and Figure 9). Regarding the frequencies of basic lymphocyte subsets, the linear tendency of NK cells and the T-NK lymphocyte proportion were otherwise significantly higher in PP and NP than in controls (Table 1, Figure 1 and Figure 5). A positive correlation was observed between age and levels of lymphocytes, B-lymphocytes, and monocytes in both PP and NP (Table 1, Figure 2, Figure 5 and Figure 7). A negative correlation was observed between PP and NP groups with regard to T-mature CD3, monocytes CD14+CD16+, CD4 naïve, and CD8 naïve (PP low, NP up) (Table 1, Figure 2 and Figure 5, Figure 6, Figure 7, Figure 8 and Figure 9). A negative correlation was also observed inside the PP group between neutrophils, T-killer, and NK cells (increased) (Table 1, Figure 4 and Figure 5) and lymphocytes, CD4, B-lymphocytes, monocytes CD14+CD16+, CD4 naïve, CD8 naïve, and monocytes (decreased) (Table 1, Figure 2 and Figure 5, Figure 6, Figure 7, Figure 8 and Figure 9) (*p* < 0.001). A negative correlation was observed between lymphocyte subsets within the NP group, neutrophils, T-mature CD3, CD4, NK cells, T-killer, monocytes CD14+CD16+, CD4 naïve, and CD8 naïve (increased) (Table 1, Figure 1, Figure 2, Figure 3, Figure 4, Figure 5, Figure 6, Figure 7, Figure 8, Figure 9, Figure 10, Figure 11, Figure 12 and Figure 13 and lymphocytes, B-lymphocytes and monocytes (decreased) (Table 1, Figure 2, Figure 5 and Figure 7) (*p* < 0.001).

## 4. Discussion

COVID-19 is a highly infectious virus that can be transmitted by air droplets, contact, and fecal–oral infection, making it not only difficult to control, but also to predict its transmission. Due to the increased risk of transmission and due to the intricate structure of the immune system, it has become extremely difficult to create a uniform therapeutic protocol valid for everyone. It follows that analysis of peripheral blood lymphocyte subsets has become a key factor to understand all of the implications of the COVID-19 disease [43]. At present, there are increasing warnings of changes in T- and B lymphocytes and their subtypes in either diagnosis or treatment. With the increase in COVID-19 research, we have witnessed some reports indicating changes in lymphocyte counts especially regarding CD4, CD8, CD4 naïve (CD45RA), and B cells. In the present study, the COVID-19 group included critically affected individuals with fever and dyspnea (positive to oral-nasopharyngeal swab/RT-PCR, CT with ground glass opacities, pO2 < 60%). The second group, named COVID-like or NP, included patients with fever and slightly dyspneic though negative to oral-nasopharyngeal swab/RT-PCR with CT ground glass opacity images. The third group, NA, consisted of patients affected by respiratory diseases of non-COVID-19 origin, negative to oral-nasopharyngeal swab/RT-PCR without ground glass opacities. The fourth group, NN, consisted of healthy individuals. In our experience, the majority of patients affected by COVID-19 shared a quite common scenario characterized by important high levels of IL-6 and CRP presented with increased serum fibrinogen and troponin with low levels of eGFR and hemoglobin and hematocrit, with slightly high levels of RDW (suggestive of iron anemia) [39]. COVID-19 seems to induce a lack of coordination between the two branches of immune system, innate and adaptive immune responses. While the innate system provides indications in the forms of chemical signals (cytokines and interleukins) or degraded bio-products of infectious organisms (antigens) to stimulate the adaptive immune responses, the latter should remain disengaged until the “antigen presentation” process takes place. The adaptive system takes time to participate before two key cell types—B cells and T cells—come onto the scene, in which pro-inflammatory, and immune modulator actors work in complete synchrony mode [40,41,42,43,44,45,46,47,48,49,50,51]. Once the adaptive immune system cells have acted on the pathogen, a pool of long-lived memory T and B cells are formed and released into the system. These memory lymphocytes remain in silent inactive mode until the system encounters the same pathogen acting much faster and with more affective immune response. Memory is the cornerstone of the adaptive immune system, allowing a prompt and long-term protection [51].

Therefore, in such a highly complicated condition it was crucial to determine the features of lymphocyte subsets in COVID-19 since they provide us information to explore in greater detail the pathophysiology of this disease. Clinically, the findings as a whole show a very peculiar picture when related to lymphocyte subsets in COVID-19 patients.

In this study, we investigated the presence of SARS-CoV-2 in 138 individuals. We analyzed B-cell, T-cells, and monocytic subsets in all subjects at the time of admission (T0) by flow cytometry analysis.

The PP patients’ immune profiles showed low levels of B-lymphocytes, low levels of T-regs CD4+CD25+, high levels of T CD8+CD38+DR, high levels of T-suppressor CD8+CD57+, high levels of T-NK CD3+CD56+ (higher than NP, NN, and NA). High levels of neutrophils, low levels of lymphocytes, low levels of T-mature CD3, low levels of CD4, low ratio < 1 CD4/CD8, low levels of CD4 naïve (lower than NP but higher than NN and NA).

The NP patients exhibited high levels of Neutrophils, NK, CD3+DR+, Monocytes CD14+CD16+, and low levels of lymphocytes, T-mature CD3+, CD4+, CD8+, low ratio <1 CD4/CD8, low levels of CD4 naïve, and CD8 naïve (higher than PP, NN, and NA). High levels of T-NK CD3+CD56+, high levels of T-suppressor CD8+CD57+, high levels of T CD8+CD38+DR, high levels of monocytes (lower than NP but higher than NN, NA).

The NN patients exhibited low levels of B-lymphocytes and neutrophils similar to those in the NP group (Table 1). Consistent with the above data, when examining the distribution across the four groups, the negative correlation between T-subset pro-inflammatory compartment and the modulatory lymphocyte compartment in relation to age range and sex profile was significant, with a clear predominance of males prevalent in both PP and NP groups (Figure 2, Figure 3, Figure 4, Figure 5, Figure 6, Figure 7, Figure 8 and Figure 9; Table 1).

The results were also statistically significant and revealed a sort of homogeneity of the data (St.dev.95%; *p* = 0.01) confirming the flow cytometry outcomes. With regard to the WBC, the trend indicated that PP patients showed the lowest level of WBC, very close to those in the NN group. The neutrophils trend in PP, NP, and NN grew with age, nevertheless, in both the PP and NA groups the neutrophil increase started from a lower range compared to NP, a range that was already at the high limit, with the highest levels seen in the older patients. The graphics show decreases in lymphocytes in PP, NP, and NN groups, however in both PP and NN the trend started from a higher range while the NP the range started significantly lower. Notably, from around 80 years of age, we observed the worst decay. The frequencies of T-mature CD3+ in COVID-19 patients, NN and NA versus NP showed a negative correlation. The levels of T mature CD3 in NP quantitatively increased without correlation to age, and the increase started from a lower level, whilst in the PP and NN patients T CD3+ declined with age, starting from higher level. Low T cell count usually indicate conditions affecting the immune system or lymph nodes due to viral infections such as influenza, and aging. A negative correlation was observed between the age and the percentage of T CD4 between PP and NP. The NA individuals showed a normal range of CD4 at all ages. The graphics of PP showed T-CD4 decline with age starting from a normal level range, conversely NP showed a rise starting from a lower range and increasing with age, the older the higher. Frequencies of T-CD8+ lineage in COVID-19 patients and controls (NP, NN and NA) showed a correlation between range level and age that can be seen in all groups. A slightly decrease in T-CD8+ in PP and NN groups (the older the lesser) and a light increase of CD8 and age in the NP individuals (the older the higher) can be seen.

Frequencies of CD4/CD8 ratio showed low quantitative trend (<1) in both the PP and NP, but not in the NN group. A low or inverted CD4/CD8 ratio is suggestive of an anomalous immune activity, immune senescence, and chronic inflammation, conditions that can be seen in HIV infection. The prevalence of an inverted CD4/CD8 ratio increases with age. Frequencies of NK CD16 lineage in COVID-19 patients and controls (NP, NN, and NA). A strong correlation was observed between age and the NK increase in the PP group the older the higher; the NP group showed a smaller increase, though the starting level of NK cells was higher compared with PP. The frequencies of B Lymphocytes in COVID-19 patients and controls (NP, NN, and NA) revealed a linearity; B-lymphocytes in PP individuals at time of the admission showed a significant quantitative low level without correlation to age, whereas both NP and NN individuals started with a higher level of B cells showing a quantitative decrease correlated with the age. The frequencies of T- and N killer CD3+CD57+ showed a quantitative increase also correlated to age in the PP and NP groups (high) and NN (low) suggestive of the active capability of immunity to respond as killer T cells recognize and kill virus-infected cells because of the viral antigen on its surface. The frequencies of CD14+CD16+ Monocyte observed in COVID-19 patients and controls (NP, NN, and NA) revealed a slight quantitative decrease in the PP group, which was not correlated with age, conversely, a quantitative increase in NP correlated with age was observed. The increased levels of CD14+CD16+ monocytes in the elderly may indicate they have undergone the senescence process and therefore they secrete proinflammatory factors in response to low-intensity antigenic stimuli. However, the levels of both intermediate CD14+ and CD16+ as well as non-classical monocyte Neutrophils and NK cells were significantly elevated in NP patients. The graphic showed a slight quantitative decrease in CD4+CD45RA naïve in the PP group without correlation to age; however, there was a quantitative increase in CD4 naïve in correlation to age in NP individuals. Of note, the starting level of CD4 naïve in the NP group was significantly lower than the one seen in PP group, though the mechanism is yet to be clarified as, unlike age related changes in other T cell subsets, the stable level of T memory cells in peripheral blood may support CD4 naïve cells for long-term immunological memory, while their importance may increase together with ageing. Higher morbidity and mortality following infections, particularly influenza, has been observed in the elderly individuals. The frequencies of CD8+/CD45RA Naïve levels showed a slight quantitative decrease in the PP group without correlation to age, while a quantitative increase in CD8 naïve in correlation to age in NP individuals was observed. Notably, CD8 naïve in the NP group started to increase from a lower range level than PP. Though the mechanism is yet to be clarified as, unlike age related changes in other T cell subsets, the stable level of T memory cells in peripheral blood may support CD8 naïve cells for long-term immunological memory, while their importance may increase together with ageing. Regarding monocytes, the results showed a decline in both PP and NP as age increased; a condition that is mainly due to a general decline in naïve lymphocytes in the bone marrow and thymus as well as the expansion of incompetent memory lymphocytes. The frequencies of T-regs in COVID-19 patients indicates a quantitative linearity without correlation to age. The NP group showed the lowest quantitative level of T-regs with a correlation to age. The NN group was observed in between the PP and NP. A prominent feature of severe COVID-19 is determined by uncontrolled pro-inflammatory cytokine activity, the so-called “cytokine storm” that is in part due to the deficiencies in immune regulatory mechanisms of T-regs, which are key regulators of immune responses and essential in the maintenance of immune homeostasis. The T-regs modulate the antiviral defense at the early stage of infection and reduce inflammation at the later stage of COVID-19. Frequencies of T-suppressor CD3+CD57+ in COVID-19 described a quantitative increase in PP and NP correlating to age. The NP group revealed the highest rate of T-suppressor in elderly patients; the T-suppressor also increase in NN but in this case there was no correlation to age, with the graphs indicating more uniformity between the individuals and age. The original function of T-suppressor is blocking the activity of some other types of lymphocytes, to keep the immune system from becoming over-active. T-suppressor CD8+CD57+ also plays a significant role in various diseases or conditions, associated with chronic immune activation such as cancer, chronic intracellular infections, and some chronic pulmonary diseases.

Thus, what should we consider as “*Immunologically ordinary in COVID19?*” As expected, both COVID-19 patients and Covid-like patients revealed a kind of homogeneity either clinically or laboratory-wise. The existing similarities between PP and NP (lesser with NN patients) may reveal a clinical picture consistent with common patterns of humoral and cellular immune responses against pathogens. The presence of differences, on the contrary, could unveil specific traits of COVID-19 disease ignored until now.

We should highlight that the NP patients, at the date of admission at 118 Unit, presented a lower level of inflammation, less dyspnea and better ABG profiles (data not shown) although more lethargic and physical exhausted, a picture that somehow recalled a post-COVID-19 phase. Though negative with RT-PCR, the NP individual laboratory parameters unveiled an evolved inflammatory state in which immune alterations were somehow more stable. The prevalence of an inverted CD4/CD8 ratio increasing with age and the presence of metabolic disorders such as DM2, hypertension, overweight, and kidney and heart diseases [28,29,33,34,35,43,45,46,52,53,54].

However, dissimilarities, more than similarities would expose the unique tropism of SARS-CoV-2 that directly affects main lymphoid organs with the ability to hide in remote areas within tissues and cells, insistently triggering the release of inflammatory mediators in an equivalent manner of any chronic lung disease. Indeed, NP patients showed a clear quantitative increase in CD8+ (that was not seen in PP and NN), a stronger decrease in both CD4/CD8 naïve cells, a marked decrease in CD4+/CD8+ ratio and stronger increase in T CD3+DR+; a scenario that opens a debate on whether there is a stage-associated pattern of these markers expression on T-lymphocytes during SARS-CoV-2 infection, since specific phenotypic patterns may have functional links in the host response to the virus [1,18,20,30,31,55,56,57,58,59,60,61,62,63,64,65].

Similarly, if we consider B-lymphocyte fluctuation between the different groups, a diminished B-cell modulatory activity would inhibit the conversion process of T cells to regulatory T cells (Tregs) generating a more inflammatory environment. This phenomenon was clearly seen in both PP and NP groups, where severe T cell depletion (regulatory phenotypes) was consistently associated with high neutrophil and NK levels, a significant sign that occurs during the first days of the onset of symptoms [30,31]. This phenomenon was clearly seen in both PP and NP groups in which, severe T-regs depletion was clinically concomitant with an increase in neutrophils and NK cells within the lungs’ interstices and parenchyma. This is a significant pattern often seen within the first days of COVID-19 symptoms onset and confirmed by the presence of bilateral ground-glass opacities by thoracic CT-scan (Table 1, Figure 5) [44,45,65,66,67,68,69,70]. This condition, widely described, was consistently characterized by the presence of lymphocytic granulomatous, viscous neutrophilic extracellular trap (NET) followed by massive and systemic thromboembolic events often associated with a unique arterial blood gas (ABG) panel, suggestive of reduced gas exchanges characterized by hypoxia and hypocapnia (low O_2_/ low CO_2_) and alkalotic pH. These parameters are the hallmarks of a silent progressive organ decay, known as “*happy hypoxemia*” [36,37,38,39].

Theoretically, these findings may open-up relevant aspects of the NP condition, as we may also refer to NP individuals as part of the “Covid-like/Long Covid” disease. The NP patients seemed to reflect post-acute phase of the COVID-19, showing symptoms and signs typical of a disease that is becoming chronic (Figure 2, Figure 3, Figure 4, Figure 5, Figure 6 and Figure 7). For instance, the increased quantitative decline correlated to age (the older the lesser) in these CD4+CD45RA naïve lymphocytes in the PP populations corresponded to a rise in the NP individuals, that showed a rise in these cells from very low level (the older the higher). Along the same line, a shift towards further activation of CD8+, CD8+suppressor, T-active CD3+DR+ cells might be also indicative of the presence of SARS-CoV-2 responsive T cells within the NP pool suggesting that NP patients might have commenced a phase of adaptive immune response. Eventually, these differences illustrated that reference intervals should be relevant to the population they serve [55,56,57,58,59].

It should be stressed that, the NP patients reported in this article were not considered COVID-19 patients and were treated either in other departments or dismissed soon after the first days of treatment in 118 Unit. Notably, at six months from the date of last negative naso-oropharyngeal swab, the majority of post COVID-19 patient were still showing signs of infection complaining of general malaise, shortness of breath, fatigue, generalized body ache and recurrent headaches. The CT scans confirmed remarkable signs of lung damage with, CBC still revealing high white cell count (WBC), low lymphocyte, high neutrophils, elevated VES, CRP, fibrinogen, hypokalemia, sideremia, high T CD3+ (%), low B-lymphocytes, high T helper CD3+ CD4+ (%), high CD4+/CD8+ ratio, and low NK CD16+ CD56+ (%). As widely reported, bronco-alveolar fluids (BALF) samples obtained from COVID-19 deceased/survived patients confirmed the presence of Sars-CoV-2, which confirmed either higher expression of ACE2 receptor in the lower part of lungs or, the migratory ability of the virus towards the lower airways [39,55,56,57].

These remarks may refer to a complex scenario in which both innate and innate-like immune cells are present in regular numbers at early stages of the disease and, might also indicate that this arm of the immune system contributes to containing early, middle, and advanced virus infection [55,56,57,58,59]. Taken together, these results may suggest the validity of these lymphocyte subpopulation measurements as additional diagnostic tools to distinguish the different steps of the disease, to differentiate its grade of severity, and to predict the possibility of silent long-term viral presence to better plan a post-disease management strategy. Secondly, the limitation of RT-PCR procedure becomes clear, that it is totally dependent on the specimen site with a high risk of false negative results [43,45,46].

However, there are many questions that remain to be clarified, such as why some individuals revealed severe symptoms and survived while others suddenly died, or why others (the majority) were positive but completely asymptomatic. It will be a necessary step determining whether these differences are due to the individual’s environment, genetic predisposition, or existing comorbidities. This procedure would require the screening for recent contracted viral infections such as TB, HPV, HV, or HBV and non-diagnosed congenital diseases which increase the susceptibility to COVID-19 infection [55,58,62,63,64,65,66,67,68,69,70].

In addition, the search for single nucleotide polymorphisms (SNPs) may unveil the involvement of specific immune regulatory genes, such as those like IL-10, IL-6, and VDR or those that instruct human cells to build the receptor ACE2 that would explain the upsurge of the uncontrolled “cytokine storm” that drives severe COVID-19 multiple organ failures and death. The association between inflammatory interleukins and cytokine hyper-expression and SNPs comes from a study performed by our team recently. The outcomes showed how chronic periodontitis, considered a complex pathology, represents a significant imbalance between pro-inflammatory and anti-inflammatory gene expression such those regulating IL-10 gene and IL-6, tumor necrosis factor alpha (TNFα), IL-1α-β-RN (IL-1α-β-RN), collagen type-l alpha (COLIA1), and vitamin D receptor (VDRs) genes [68,69,70,71,72,73,74].

At the time of this study, the Fall 2020 serological analysis was not really an opportunity in hospitals; our 118 unit was an emergency unit based on fast acceptance and rescue, fast screening (RT-PCR), CBC, CT scan, etc., all of which had to be done quickly and with high precision. Based on these evaluations, the low B-lymphocytes seen in both NP and PP groups made us highly suspicious of the validity of antibody screening levels to determine a clear distinction between COVID-19 patients and the others, as low B-cells is always equal to low antibodies. Therefore, cell mediated immunity as described above remains the best criteria to sort out the dilemma. Could we consider the NP group to be a RT-PCR false-negative cohort? Probably yes; some of them they were possibly false-negative. However, the majority showed clear signs of a lung infection highly similar to SARS-CoV-2 infection, with ground-glass lungs, neutrophils and lymphopenia, low-B cells, difficulty in breathing, etc. At that time no-one knew what was really going on and, the key point to assess COVID-19 positivity was exclusively based on RT-PCR result. NOtably, as stated above, we experienced negative RT-PCR patients that were eventually admitted into ICU and only when BALF was screened did they test positive to COVID19. To conclude, we are currently visiting post-covid patients and the situation is everything but normal, even 8–10 months after the disease. The long-covid and covid like patients reveal something that must be related to COVID19 since none of them has revealed to be affected by another disease. Current literature has also started showing similar conclusions, which explain the unique tropism of the SARS-CoV-virus’s ability to run and hide in unthinkable locations without giving an evident sign of its presence [75,76,77]. Therefore, who may tell or deny that during the fall 2020 variants were already there? Why were BALF tests positive and RT-PCR tests negative?

## 5. Conclusions

We are aware of the limitations of the current study. This is a retrospective work, and it was conducted in a single hospital, which resulted in low numbers making the findings limited by the sample size. In addition, the lack of specific reagents to be used in flow-cytometry procedures should be considered a strong limitation. Nevertheless, it included patients that were transferred from large sub-urban areas that witnessed thousands of infected individuals during the pandemic. Despite the small sample size and lab limitations, different researchers have observed similar phenomena while this manuscript was under consideration. At the time of admission, the patients did not have their laboratory results and the entire clinical and laboratory measurements regarding lymphocyte at disease onset were therefore randomly obtained at that time. Regardless of these limitations, the present study, to the best of our knowledge, is one of the few investigations to examine changes in lymphocyte subpopulations in four different groups: COVID-19, Covid-like, general pulmonary patients, and healthy individuals. We found that COVID-19 and Covid-like are most likely two aspects of the same disease suggesting that monitoring lymphocyte subpopulations could be of clinical significance in the diagnosis and management for COVID-19 patients.

## Figures and Tables

**Table 1:** Flow-cytometry summary and statistical profile of four groups included in this study (CI 95%, conf.δ). The frequency of T cell subsets and B-lymphocytes were analyzed from blood collected in COVID-19 patients (PP); NP (negative swab-CT ground glass); NN (patients with different lung pathologies); NA (healthy individuals). NV% = normal value (%); St.Dev = standard deviation; Conf. δ = confidence delta. The red color indicates the worst level and the green the second worst; Norm: Normal.

SubSet NV%	Neut 45–75	Lym 20–45	Tmat CD3 61–84	CD4 25–65	CD8 15–50	CD4/CD8 Ratio 1.0–4.0	NK CD16+/CD56+ 4–28	B Lym 10–40	T-NK CD3+CD56+ <5	T-CD3+DR+ Active 1–18	CD8 Cyt. 1–28
**PP**	49%	64%	31%	20%	7%	26.67%	26.67%	60%	73.3%	13.3%	0%
**N. 45**	22 ≥ 75	27 ≤ 20	14 ≤ 61	9 ≤ 25	3 ≤ 15	12 ≤ 1	12 ≥ 28	27 ≤ 10	33 ≥ 5	6 ≥ 18	Norm
**St.Dev**	15.20	10.95	13.82	12.68	11.10	0.85	12.14	6.12	9.57	11.41	8.17
**19.42**											
**Mean**	74.85	17.07	66.38	36.38	27.03	1.59	22.26	10.16	10.98	11.70	15.10
**62.33**											
**CI 95%**	4.56	3.29	4.15	3.81	3.33	0.25	3.65	1.84	2.87	3.43	2.45
**Conf. δ**											
**NP**	78.4%	91.9%	40.5%	43.2%	18.9%	43.2%	35.1%	43.2%	67.6%	45.9%	0%
**N.37**	29 ≥ 5	34 ≤ 20	15 ≤ 61	16 ≤ 25	7 ≤ 15	16 ≤ 1	13 ≥ 28	16 ≤ 10	25 ≥ 5	17 ≥ 18	Norm
**St.Dev**	17.93	8.22	13.87	12.45	14.89	1.28	12.93	11.87	8.43	10.13	7.29
**16.67**											
**Mean**	80.41	9.68	62.24	29.98	28.90	1.54	22.68	14.36	10.52	17.89	15.15
**75.41**											
**CI 95%**	5.97	2.73	4.61	4.14	4.95	0.42	4.30	3.95	2.80	3.37	2.42
**Conf. δ**											
**NN**	45%	52.5%	17.5%	5%	17.5%	17.5%	10%	42.5%	65%	17.5%	0%
**N.40**	18 ≥ 75	21 ≤ 20	7 ≤ 61	2 ≤ 25	7 ≤ 15	7 ≤ 15	4 ≥ 28	17 ≤ 10	26 ≥ 5	7 ≥ 18	Norm
**St.Dev**	13.75	10.76	10.21	11.67	9.38	1.18	10.03	5.39	4.76	7.19	6.27
**17.93**											
**Mean**	70.99	19.34	70.86	43.42	24.66	2.1	17.19	11.18	7.49	11.58	14.76
**58.5**											
**CI 95%**	4.39	3.43	3.26	3.73	2.99	0.37	3.20	1.72	1.52	2.29	2.00
**Conf. δ**											
**NA**	0%	0%	6%	0%	0%	0%	12.5%	12.5%	62.5%	0%	0%
**N. 16**	Norm	Norm	Norm	Norm	Norm	Norm	2 ≥ 28	2 ≤ 10	10 ≥ 5	Norm	Norm
**St.Dev**	5.17	4.75	8.13	5.58	4.56	0.41	7.75	3.01	12.03	3.46	5.37
**8.41**											
**Mean**	55.81	32.67	74.18	44.16	26.13	1.74	13.3	12.36	9.29	7.96	18.36
**42.6**											
**CI 95%**	2.75	2.53	4.33	2.97	2.43	0.21	4.12	1.60	6.41	1.84	2.86
**Conf. δ**											
**SubSet** **NV%**	**CD8 suppr CD57+** **0–10**		**CD8+CD38+** **DR+** **0.30–2.30**		**T-reg CD4+CD25+^high^** **< 10**	**CD4/CD45RA Naïve** **26–62**		**CD8/CD45RA Naïve** **16–40**	**MONO** **16–40**	**MONO CD14+/CD16+** **1–10**	
**PP**	64.44%		80%		37.78%	33.33%		53.33%	28.89%	17.78%	
**N. 45**	29 ≥ 10		36 ≥ 2.30		17 ≤ 10	15 ≤ 26		24 ≥ 40	13 ≥ 10	13 ≥ 10	
**St.Dev** **19.42**	7.21		11.97		14.33	19.94		22.00	3.84	6.53	
**Mean** **62.33**	12.00		8.52		13.64	36.42		42.52	8.06	6.43	
**CI 95%** **Conf. δ**	2.16		3.59		4.30	5.99		6.61	1.15	1.96	
**NP**	62.16%		51.35%		18.92%	48.65%		73.84%	18.92%	32.43%	
**N. 37**	23 ≥ 10		19 ≥ 2.30		7 ≤ 10	18 ≤ 26		14 ≥ 40	7 ≥ 10	12 ≥ 10	
**St.Dev** **16.67**	10.12		16.57		4.06	13.62		19.56	4.35	6.47	
**Mean** **75.41**	14.34		7.65		6.41	24.84		32.60	6.06	8.30	
**CI 95%** **Conf. δ**	3.37		5.51		1.35	4.53		6.51	1.45	2.15	
**NN**	37.5%		45%		25%	32.5%		55%	17.5%	13.5%	
**N. 40**	15 ≥ 10		18 ≥ 2.30		10 ≤ 10	13 ≤ 26		22 ≥ 40	7 ≥ 10	13 ≥ 10	
**St.Dev** **17.93**	5.62		2.43		14.66	18.32		15.66	3.62	6,45	
**Mean** **58.5**	9.89		2.23		11.26	37.4		44.19	7.4	8.12	
**CI 95%** **Conf. δ**	1.79		0.77		4.68	5.85		5.00	1.15	2.06	
**NA**	12.5%		18.75%		50%	0%		56.25%	12.5%	18.75%	
**N. 16**	2 ≥ 10		3 ≥ 2.30		8 ≤ 10	Norm		9 ≥ 40	2 ≥ 10	3 ≥ 10	
**St.Dev** **8.41**	5.42		2.59		14.45	11.84		15.27	1.51	3.11	
**Mean** **42.6**	7.88		1.99		15.86	32.66		39.23	7.66	7.21	
**CI 95%** **Conf. δ**	2.89		1.38		7.70	6.31		8.13	0.80	1.66	

## Data Availability

All experimental data to support the findings of this study are available by contacting the corresponding author upon request. The authors have annotated the entire data building process and empirical techniques presented in the paper.
